# Alignment-free inference of hierarchical and reticulate phylogenomic relationships

**DOI:** 10.1093/bib/bbx067

**Published:** 2017-06-30

**Authors:** Guillaume Bernard, Cheong Xin Chan, Yao-ban Chan, Xin-Yi Chua, Yingnan Cong, James M Hogan, Stefan R Maetschke, Mark A Ragan

**Affiliations:** 1 University of Queensland, Institute for Molecular Bioscience, St Lucia, Brisbane, Queensland, Australia; 2 University of Melbourne, School of Mathematics and Statistics, Parkville, Melbourne, Victoria, Australia; 3 QFAB Bioinformatics, Queensland Cyber Infrastructure Foundation Ltd, St Lucia, Brisbane, Australia; 4 Institute for Molecular Bioscience, St Lucia Queensland, Australia; 5 Queensland University of Technology, School of Electrical Engineering and Computer Science, Brisbane, Queensland, Australia; 6 IBM Research Australia, Carlton, Melbourne, Victoria, Australia; 7 University of Queensland, Institute for Molecular Bioscience, St Lucia, Brisbane, Australia

**Keywords:** alignment-free, phylogenomics, lateral genetic transfer, k-mer, D2 statistics, TF–IDF

## Abstract

We are amidst an ongoing flood of sequence data arising from the application of high-throughput technologies, and a concomitant fundamental revision in our understanding of how genomes evolve individually and within the biosphere. Workflows for phylogenomic inference must accommodate data that are not only much larger than before, but often more error prone and perhaps misassembled, or not assembled in the first place. Moreover, genomes of microbes, viruses and plasmids evolve not only by tree-like descent with modification but also by incorporating stretches of exogenous DNA. Thus, next-generation phylogenomics must address computational scalability while rethinking the nature of orthogroups, the alignment of multiple sequences and the inference and comparison of trees. New phylogenomic workflows have begun to take shape based on so-called alignment-free (AF) approaches. Here, we review the conceptual foundations of AF phylogenetics for the hierarchical (vertical) and reticulate (lateral) components of genome evolution, focusing on methods based on *k*-mers. We reflect on what seems to be successful, and on where further development is needed.

## Introduction

Phylogenomics refers to an important body of theory, methodology and tools applicable to the comparative analysis of genome-scale data within an evolutionary context [1–4]. The field builds on molecular phylogenetics, which since the early 1960s has been developed to elucidate genealogical relationships and evolutionary processes within families of genes or proteins. As the first area of molecular bioscience to develop an explicitly algorithmic approach, and drawing richly on statistics and computational science, phylogenetics is considered a major area within bioinformatics.

By definition, the inference of genealogical relationships must be based on homologous elements. Even before molecules could be fully sequenced, it was known that certain oligopeptides were common to representatives (in different biological species) of individual proteins, e.g. α-haemoglobin or insulin; similarly, 16S ribosomal RNAs in different species shared sets of short oligonucleotides [5]. Indeed, the presence of shared identity in sequences beyond the extent required to deliver conserved function was taken as evidence for homology [6]. As full-length sequences became available, it made sense to discover and display these conserved regions in a multiple sequence alignment (MSA) [7, 8]. Molecular phylogenetics is thus endowed with a richness and precision rarely seen with phenetic characters: homology is no longer ‘overall’ and subjective, but can be evidenced column by column along a set of aligned sequences. Thus, an MSA is an explicit position-by-position hypothesis of homology. Not coincidentally, an MSA matrix serves as a convenient input to software programs that calculate pairwise dissimilarities (for distance methods) or compute a tree that best explains the patterns in the aligned columns, given a model of sequence change over time (in e.g. parsimony or likelihood methods).

With the advent and spread of genomics, large data sets have become available for phylogenetic inference, with sequences longer (genomes rather than single genes or proteins) and much more numerous. It is no longer unusual to encounter data sets with thousands of genome or (concatenated) exome sequences. Phylogenomics at this scale requires organized data management, significant computational power and large memory. However, phylogenomics can present challenges other than those arising purely from size and scale: the data may be of low quality, assembly may be poor or nonexistent, the sequences may not be collinear over their entire length, different models of sequence change probably apply (e.g. to protein-coding and noncoding regions) and sequence regions may have different origins and evolutionary histories [9]. We consider these factors in turn.

## Classical (alignment-based) phylogenetics

In the early days of genomics, much effort went into ‘finishing and polishing’—joining contigs and resolving conflicts to recover full-length chromosomes nearly free of ambiguities or errors. Thanks to this body of earlier work, key information (e.g. presence or absence of a gene or pathway) can now often be obtained simply by deep sequencing followed by a rough assembly. Depending on one’s scientific goals, the breadth-versus-depth trade-off can be pushed dramatically towards breadth, at the expense of data quality. Survey projects now target tens of thousands of bacterial genomes, few of which will assemble into a single contig, while large eukaryote genomes can be approached through transcriptomics, with consequences for MSA including the need to deal with truncated sequences and alternative splice forms. Indeed, there is optimism that phylogenetic trees might be inferred entirely without assembly [10].

Basic MSA requires sequences to be collinear, i.e. to preserve a common ancestral order of elements. Depending on the sequences being compared, these elements may be, for example, nucleotides, codons, amino acids, domains, exons or genes. Non-collinearity may arise because of poor sequence quality or misassembly, but could also be real, particularly at whole-genome scale. Across bacterial genomes, gene order tends to be poorly conserved except among close relatives; exceptions include ribosomal RNA operons and some genes encoding ribosomal proteins. Thus, even with correctly assembled genomes, a separate bioinformatic step is required to match putatively homologous regions before, or as part of, MSA. Like MSA software, whole-genome aligners take different algorithmic approaches and implement different assumptions and trade-offs [11, 12] but in general are CPU- and memory-intensive, with considerable scope for error and ambiguity arising, e.g. from families of repetitive elements, low-complexity regions and paralogs.

Most approaches to phylogenetic inference require a statistical model of sequence evolution [13]. It is not difficult to imagine that different classes of sequence (e.g. those encoding a protein, a functional RNA or no product at all) are best described by different models. Even within a single gene, inference quality may be improved by applying different rate classes or steady-state assumptions, e.g. for DNA regions that encode highly structured versus unstructured regions of proteins, or stems versus loops of ribosomal RNAs. It is computationally onerous to identify, delineate and group these regions, match each to the best model and optimize parameter values. Scalability to genome-scale data would be facilitated by simplifying these models, using a single generic model or, if possible, eliminating them altogether.

In the standard Darwinian model, genomes are inherited vertically from one generation to the next within lineages. To a first approximation, this adequately describes the evolution of nuclear genomes of morphologically complex organisms including animals and plants. However, genomes of bacteria, archaea, protists, viruses and plasmids often contain stretches of DNA acquired laterally from unrelated organisms, or from the environment. Many studies indicate that 10–40% of the genes in some bacterial genomes, and essentially all gene families in bacteria, have been affected by lateral (horizontal) genetic transfer (LGT or HGT) ([14, 15] and references therein). For such genomes, a phylogenomic workflow must distinguish vertical from lateral signal, and treat each separately. To complicate matters, neither genes nor domons (genomic regions corresponding to protein domains) are privileged units of LGT [16, 17]; new lateral events can overwrite older ones; regions of lateral origin may ameliorate, i.e. evolve to become indistinguishable from their new host genome [18]; and older lateral regions will themselves be inherited vertically within subtrees [15, 19]. In MSA-based phylogenomics, these issues are addressed by adding further (computationally demanding) steps to the workflow, e.g. inferring an organismal reference tree and comparing features of its topology with those of individual gene or protein family trees [14, 20]. Opportunities abound for complications to arise from cryptic paralogy, or inappropriate delineation of the units of analysis.

Based on the above, we might summarize our wish list for next-generation phylogenomics [9]: it must be based on homologous signal (a different subset of signal for each evolutionary origin), while avoiding the assumptions inherent in MSA (predefined fixed units of analysis; colinearity). It should incorporate a generic, computationally simple substitution model; be highly scalable to large data, yet robust to low data quality; and perhaps support phylogenetic inference from unassembled sequence reads. Alignment-free (AF) methods offer considerable promise against each of these goals [9].

## Alignment-free methods and *k*-mers

AF methods underpin key algorithms in diverse areas of bioinformatics including database searching [21, 22], sequence clustering [23], error correction in sequencing reads [24], genome assembly [25], discriminative prediction of regulatory variants [26, 27] and testing for genetic recombination [28]. In phylogenetics and phylogenomics, AF methods offer alternatives to the assumptions and computational demands of MSA identified above. Following Haubold [29], AF methods can be classified broadly into those based on word (*k*-mer) count, and those based on match length. Certain other AF methods may fit uncomfortably into these classes, or lie outside them altogether [30]. In the present context, the motivating concept is the same: substrings (perhaps defined by *k*-mers) that meet certain criteria, and are shared by a set of sequences, can be considered as capturing part of the homology signal and are thus potentially informative on phylogeny. Here, we focus primarily on *k*-mer count methods.

Substrings (sub-sequences) of defined length are variously known as words, *k*-mers or *n*-grams, with *k* or *n* denoting the substring length. By disallowing mismatch, degeneracy and indels, *k*-mer statistics become simpler and the computation more efficient. These strictures may be slightly relaxed (e.g. by allowing limited mismatch to deal with noise) or avoided in part (by recoding into a reduced alphabet), albeit at the risk of crossing into the realm of pattern or motif analysis, for which different and computationally less-favourable methods are required.

**Figure 1:**
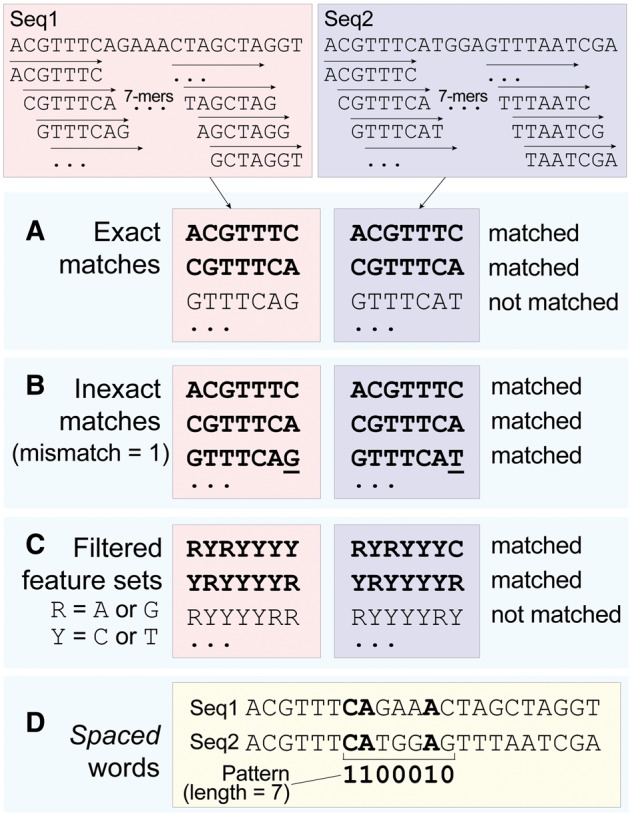
Fundamental concepts and nomenclature of *k*-mers, illustrated here for overlapping *k*-mers (*k *= 7, stride = 1) in two DNA sequences. (**A**) Exact matches, (**B**) inexact matches, (**C**) degenerate bases and (**D**) a binary pattern of match and non-match positions (spaced word matches).

Any molecular sequence can be represented as the set of its constituent *k*-mers (Figure 1). These *k*-mers are typically allowed to overlap with stride = 1; a larger stride ≤*k* could be used to reduce the computational effort. Whereas in MSA the linear order of sequence elements is fundamental to recognizing conserved (homologous) positions and identifying conservation profiles, the analogous concept in AF is an order-less matching of *k*-mers, i.e. an intersection of *k*-mer sets. For sufficiently large *k*, any given *k*-mer is approximately unique to a sequence [31], so in the absence of extenuating circumstances (e.g. strong mutational bias or low-complexity regions), shared instances of that *k*-mer are likely to be homologous. As sequences progressively diverge on a tree, they share fewer *k*-mers in common, and the longest *k*-mer they share tends to be shorter. As we discuss below, these measures can be used to estimate a pairwise distance. For this, the count or frequency of shared *k*-mers seems to be sufficient, i.e. it is not necessary to keep track of positional information [32] unless we wish to map specific *k*-mers (e.g. those inferred to have a lateral origin) to genes, structures or functions [33–35].

In contrast, conservation profiles measure local sequence similarity and thus require at least approximate positional information. Classical alignment algorithms such as Smith–Waterman or Needleman–Wunsch use dynamic programming to determine columns and/or blocks of matching residues in a set of sequences. From the number or proportion of conserved residues within a column, a conservation profile can be derived. AF conservation profiles can be constructed by plotting the maximum number of matching *k*-mers over their mean positions within the set of sequences [36]. While AF conservation profiles do not achieve single-residue resolution, they are faster to compute than classical profiles (linear versus quadratic time with respect to sequence length), yet still allow the identification of conserved regions or domains, even if non-collinear [36].

Extraction of *k*-mer sets from molecular sequences is in principle trivial, accomplished simply by sliding a window of size *k* over a string representation of the sequence to produce a lexicon of overlapping *k*-mers (Figure 1). In application to DNA, it is usual to record only canonical *k*-mers (the lexicographically smaller of a *k*-mer and its reverse complement), or to work with only the forward strand. Efficient accumulation of *k*-mer counts requires that we determine the novelty of each *k*-mer as it appears: previously encountered *k*-mers must be identified rapidly and their counts incremented, while novel *k*-mers must be inserted quickly and without impairing the performance of the data store during subsequent queries. Approaches to this task may be categorized broadly into those based on hashing, and those relying more directly on data structures invented for string lookup and spelling correction, notably the suffix tree [37] and the suffix array [38].

Exact hashing methods offer constant-time insertion and lookup, and their use in bioinformatics has a long history. Naïve hashing, however, proves surprisingly slow [39] and requires memory linear in the number of distinct *k*-mers, which in turn is exponential in the size of the *k*-mers: $O\left({\left|A\right|}^{k}\right),$ where |A| is the size of the alphabet. Jellyfish [39] overcomes many of these problems through careful design of a lock-free multithreaded hash table, using a key encoding and bit packing to ensure far lower memory usage. Tessel, part of the Blue read-correction package [40], markedly reduces memory requirements for sequence reads by excluding singleton *k*-mers until a second occurrence is observed. Even for modest coverage, genuine *k*-mers will occur many times, with singletons almost certainly the result of sequencing error. Confirmed *k*-mers are recorded in optimized, partitioned lock-free hash tables, with a subsequent merge phase to ensure accurate final counts. Melsted and Pritchard [41] address memory usage via a Bloom filter [42], a probabilistic distributed hashing scheme that admits a small chance of a false-positive match. A standard hash table is used to store entries seen twice or more, but the implementation remains single-threaded and is not competitive. Hybrids of this nature have been used recently in the context of de Bruijn graphs [43].

Suffix trees represent a string through its underlying suffixes, each encoded as a path from the root to a leaf, with the start position of the suffix within the string stored in this terminating node. Suffix arrays contain these same start positions, but arranged according to the lexicographical order of the suffixes included. For both, construction time and space are linear in sequence length, hence in the number of *k*-mers, but the array requires far fewer bits per suffix, perhaps one-fifth to one-third of the footprint of the tree [38]. Lookup is linear in the length of the query, here $O\left(k\right).\;$Suffix trees have long been applied in substring matching (e.g. in MUMmer 1.0: [44]), while *k*-mer counters based on suffix arrays have included Meryl (part of the Celera Assembler) [45] and Tallymer [46], the latter enhanced by storing the longest common prefix among suffix groups. While the optimized hashing methods discussed above appear superior for general *k*-mer counting and retrieval, exact hashing does not preserve locality, limiting its utility for applications based on approximate matching. In contrast, suffix and tree-based approaches may preserve structure common across many *k*-mer entries, supporting mismatch neighbourhoods and correction of a letter (or longer segment) through replacement by a more strongly weighted alternative. Even so, these tasks may be prohibitively expensive for very large data sets, which may be handled more generally through careful inclusion of disk or solid-state drives.

## Phylogenetic inference based on *k*-mers

As we have mentioned, as sequences diverge over time from a common ancestor, they will come to share fewer, and shorter, *k*-mers. More precisely: given a threshold τ such that *k*-mers of length *k* ≥ τ occurring in related sequences can be considered homologous, as these sequences diverge (a) for a fixed *k* ≥ τ, the number of shared *k*-mers will tend to decrease, (b) over all *k* ≥ τ, the mean length of shared *k*-mers will tend to decrease and (c) the longest shared *k*-mer will tend to be shorter. Measures that capture these trends behave as pairwise similarities, and like their classical MSA-based counterparts can be used in distance analysis to generate a tree [47–55]. The optimal τ is likely to be problem- and data-dependent (see below), but could be selected based on the distribution of match lengths in simulated sequences [56], e.g. to maximize the area under the receiver operating characteristic curve or ensure a minimum desired frequency of true positives.

The best-known measure of *k*-mer distance is based on the *D_2_* statistic [21, 48, 57–60]. Building on a proposal by Blaisdell [61], *D_2_* is simply the count of exact *k*-mer matches between two sequences, summed over all *k*-mers at a given *k*. As this count depends on the sequence lengths, *D_2_* is often normalized by the probability of *k*-mer occurrence, or by assuming a Poisson distribution [48, 62]. Chan *et al.* [63] introduced a neighbourhood variant. Even so, for *D_2_*-based measures to be applied confidently, particularly in the comparison of closely related sequences, understanding the *k*-mer structure of actual genomes would be highly desirable [58, 64–67].

**Figure 2:**
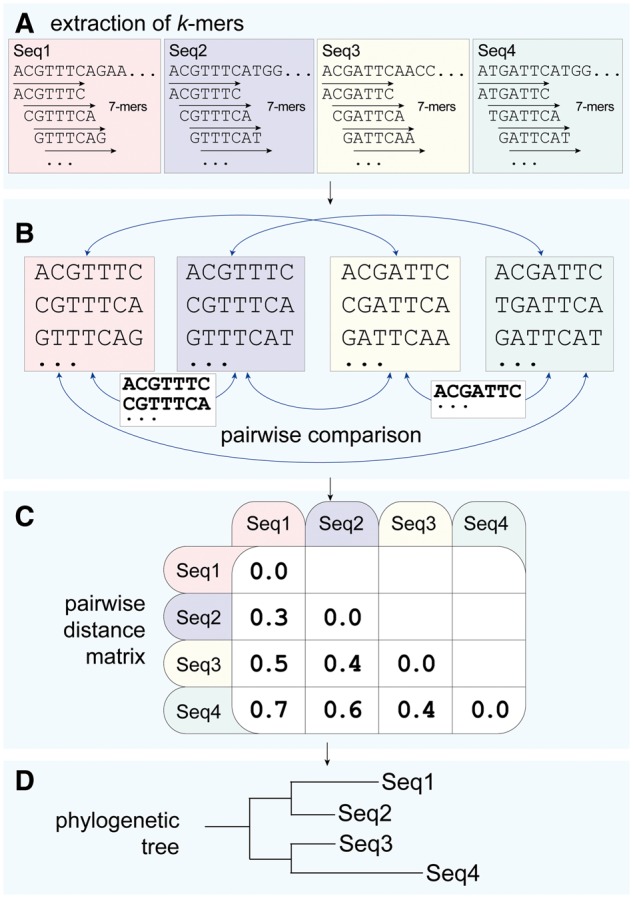
An AF phylogenetic workflow in which (**A**) *k*-mers (*k* = 7, stride = 1) are extracted from four sequences (Seq1 through Seq4), (**B**) shared 7-mers are identified by pairwise comparisons, (**C**) a pairwise distance matrix is calculated, from which (**D**) a tree is computed using a distance-based method, e.g. neighbour joining.

Bioinformatic workflows leading to AF trees differ little from their classical counterparts, except that MSA is not required (Figure 2). Putatively homologous sequences (e.g. genomes) are assembled and quality checked, e.g. for illegal characters. *K* is selected (see below), *k*-mers are extracted, and distances are computed pairwise (above) and assembled into a triangular matrix that is input into software that implements neighbour joining [68, 69] or another distance-based algorithm. Because distance algorithms build trees by clustering sequences rather than by estimating a measure of changes along internal edges, some authorities consider them non-phylogenetic. Here, we follow Felsenstein ([70]: 145–6) in relegating this distinction to debates over classification, and for the purpose at hand accept distance as a legitimate basis for the statistical inference of phylogeny. Höhl and Ragan [71] pointed out that shared *k*-mers could be arranged into a (very local) ‘alignment’ matrix and used as input into likelihood, Bayesian or other (non-distance) algorithms for tree inference, although at the cost of the speed and scalability we hoped to secure by taking an AF approach in the first place.

*K* is the critical parameter in AF phylogenomics. As we depend on *k*-mers to capture homology signal, the value we select for *k* must be large enough to ensure that few *k*-mers are present in our analysis purely by chance, but not so large that informative *k*-mers are arbitrarily excluded and signal unnecessarily attenuated. The factors most important for selecting an optimal *k* are the alphabet (e.g. nucleotide versus amino acid) and the complexity, divergence and length of the sequences under investigation. Given the complexities of sequence evolution, the performance of AF methods is best assessed by computational simulation rather than analytically. Using *evolver* in PAML [72], we simulated the evolution of sequences on a tree under a general time-reversible model to examine how G + C content, length of terminal or more-basal branches, rearrangement, truncation and among-site rate heterogeneity affect precision and recall. We also simulated indels, and explored trees generated under coalescent and non-ultrametric models [63]. Our results indicate that for *k* within an optimal range, many AF methods can perform well under basic scenarios [5, 63, 73], indeed better than MSA in the presence of rearrangement or indels [63].

In the case of empirical data, the true tree is unknown, making it impossible to assess performance using measures of precision and recall. To compare AF methods under scenarios of sequence divergence, rearrangement, inversion and LGT, we focused instead on sensitivity to change of parameter value (e.g. *k*), and accuracy in the sense of recovering accepted subtrees [73]. All nine AF methods we examined were robust against complex genome rearrangements or inversions, and most word-count methods were robust and computationally efficient against moderate levels of LGT. Performance varied with the extent of divergence, with the word-count methods more accurate than match-length methods at higher divergence. The optimal size of *k* was sensitive to the extent of sequence divergence, but was little affected by the other scenarios we simulated. Thus, for data sets of known divergence, AF methods might be applied without exploratory tuning of *k*, and could be expected to perform as well or better than MSA-based approaches. However, AF methods have not been rigorously examined under fully realistic scenarios in which different lineages may evolve at different or variable rates, under different models of substitution, and/or with biases that give rise to compositional convergence.

For large data sets of bacterial and archaeal genomes, we inferred biologically realistic AF trees in which many clades familiar from MSA-based studies were recovered. Most differences between the AF and MSA trees involved terminal branches, i.e. the most-closely related genomes. We investigated a multiple-*k* approach in hopes that longer *k* might provide better resolution at the termini, while shorter *k* would be more appropriate for the eroded signal at more-basal bipartitions. In our hands this was unsuccessful, but an adaptive or multiple-*k* approach might bear more-systematic reinvestigation. Some AF methods can also be used directly on large high-throughput sequencing data, i.e. sets of reads or contigs with only basic assembly, or none at all [10, 54].

Given sets of *k*-mers from individual sequences, the time required to compute AF distances typically scales linearly with the number of sequences; weighting, normalization or extension to inexact matches will incur additional cost [50, 52, 54, 56, 62, 73–75]. Using $D_{2}^{S}$, we could generate accurate trees for thousands of bacterial genomes in some tens of hours on a moderate-sized cluster [76]. Memory is the main limitation for *k*-mer-based approaches, but the actual demand depends on the implementation used, and can sometimes be traded off against speed of computation. AF methods with optimal memory consumption are slower than the more memory-greedy methods, with current hashing-based implementations limited to *k* = 32 in most cases [47, 50].

AF methods nonetheless retain certain limitations. In simulations, the *D_2_*-based methods we examined recover the reference topology when applied to sequences of length of 10 000 nt (e.g. small genomes; operons), but are prone to errors at 1500 nt (genes) and fail at 250 nt (domons) [63]. By disregarding singleton *k*-mers (i.e. erroneous reads), it is possible to improve distance estimates at higher coverage, but this degrades the signal at lower coverage [10]. In the MSA context, distance methods are criticized for reducing the pairwise comparison between sequences to a single number, in the process losing information on patterns of conservation within and among sequences; this is true of *k*-mer distances as well. Alternative approaches might involve a *k*-mer substitution model, but this scarcely seems feasible if the substitution matrix would be high dimensional, sparse and dependent on immense data for parameterization. Indeed, we suspect that such an approach would be so computationally expensive that it would negate the advantages of taking an AF approach in the first place. Methods exist for computation with sparse matrices, but to our knowledge have not been explored in a phylogenetic or phylogenomic context.

## Alignment-free approaches to lateral genetic transfer

For phylogenomic analysis of genomes potentially affected by LGT, we must also identify and deconvolute vertical and lateral signal. In MSA-based phylogenomics, this is done by appending a filter to the standard workflow: trees inferred for individual gene families are compared with a reference topology, and well-supported but conflicting bipartitions are taken as *prima facie* evidence of LGT [77, 78]. The corresponding gene or protein family might then excluded (to purify the vertical signal) or analysed separately to understand the sources, recipients, processes and impact of LGT.

At first glance, there is much to recommend a similar workflow for AF phylogenomics. An approach built on *k*-mers might liberate us from having to take genes, or any other predefined features, as the units of analysis. In MSA-based phylogenomics, incongruent signal can be traced back to the underlying gene-family MSA, but this does not tell us which of the aligned gene(s) is/are responsible for the incongruence, the number and quality of alternative signals or the number, quality or location of recombination breakpoints [16, 79]. AF methods might give us fine-scale access to some or all of this information. Indeed, with AF, we have further options. A genomic region might have arisen by LGT if, in the absence of extenuating circumstances, it (a) unexpectedly shares *k*-mers with a distantly related genome, and hence (b) exhibits an anomalously short *D_2_* distance to that genome. This is why (c) a distance tree computed for that region will be topologically incongruent with that computed for a vertically inherited regions, or a trusted reference tree. Interestingly, these lines of evidence exactly parallel the three main strategies for LGT detection [80–82].

**Figure 3:**
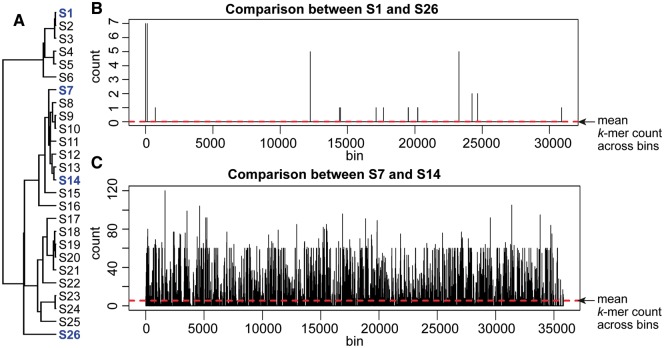
A sliding-window approach of *k*-mer sharing between sequences, illustrated here using a set of 26 sequences simulated [84] on the tree (**A**) depicted at the left. Pairwise comparisons are shown for (**B**) two highly dissimilar sequences, S1 and S26, and (**C**) two similar sequences, S7 and S14. Each plot shows the number of matching 21-mers within a 60-nt window, as it is incremented along S1 or S7, respectively.

To begin to explore these AF approaches, we simulated the evolution of DNA sequences on a tree using ALF [83] or EvolSimulator [84], and then counted how many 21-mers are shared pairwise within a sliding window of length 60 nucleotides. In the first instance, we did this in the absence of simulated LGT, so as to establish a baseline against which lateral regions could later be detected [85] (Chua, Maetschke and Ragan, unpublished). This is a *k*-mer variant of approaches long used to find lateral regions within sequences based on anomalous G + C content, dinucleotide frequencies or codon usage [18, 81, 82, 86–92]. We found that while the most-divergent sequences shared very few 21-mers (zero in most windows), a few windows shared as many as seven; although these are false positives, we could find no objective statistical criterion by which a hypothesis of LGT could be rejected for them. Conversely, with more-closely related sequence pairs, most windows shared many 21-mers, but the variation was such that it would be impossible to recognize or bound a truly lateral region (Figure 3). Note also that a sliding window approach could work only on assembled genomes or large scaffolds, not on masses of raw reads. The idea seemed promising, but something critical was missing.

Thanks to cross-disciplinary collaboration, we soon discovered what was missing. Document analysis involves concepts that can be identical or analogous to those in molecular phylogenetics [93–96] including the ‘contamination’ of texts by lateral transfer [97, 98]. A statistic known as term frequency–inverse document frequency (TF–IDF) is widely used to determine the importance of a word in a collection of documents: words that appear frequently in a document, but rarely in the rest of the corpus, carry greater importance for that document. A variant of TF–IDF might be used to detect lateral regions in molecular sequences. *K*-mers can be seen as analogues of words (albeit ones that sometimes overlap each other), groups of similar sequences as documents and a sequence database as a corpus. Unlike in a classical MSA-based workflow, sequences must be arranged into groups, but subsequent steps are AF. Sequence regions that contain *k*-mers infrequent in their own group (TF) but frequent in another group (IDF) are inferred as instances of LGT from the donor group to the recipient sequence [33]. Our unsuccessful idea above (Figure 3) represented IDF without proper TF.

**Figure 4:**
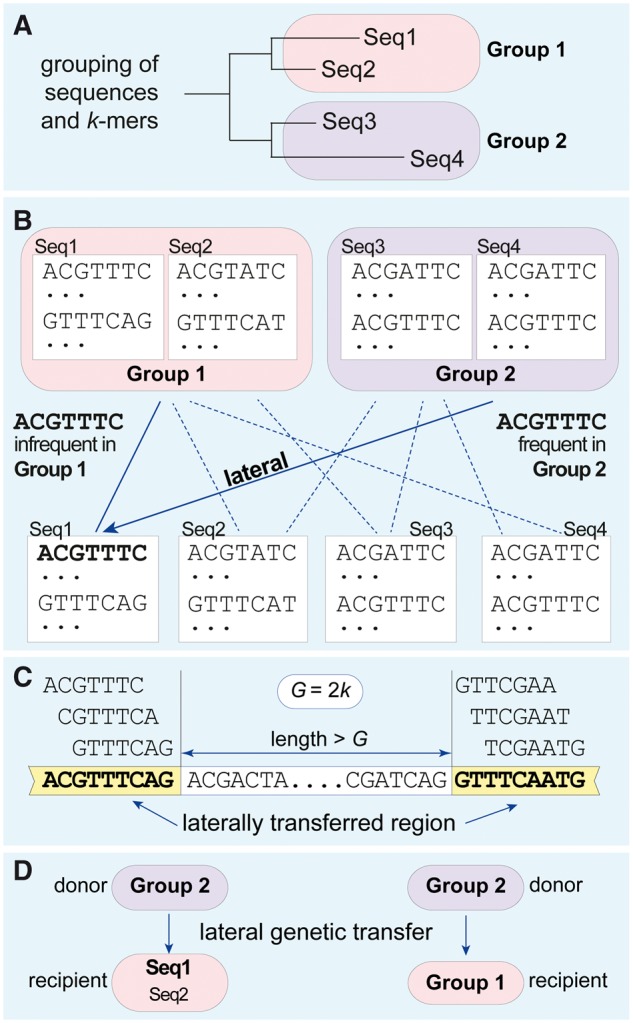
Simplified workflow illustrating the use of TF–IDF to identify lateral genetic transfer. (**A**) Four sequences (Seq1 through Seq4) are grouped, here into two groups (Group 1 and Group 2) based on a reference tree. (**B**) All *k*-mers (*k *= 7, stride = 1) from each sequence are compared against the *k*-mers found in each of the two groups. A *k*-mer that is infrequent in the group to which the sequence belongs (TF), but frequent in another group (IDF), illustrated here by ACGTTTC in Seq1 that is infrequent in Group 1 but frequent in Group 2, is inferred to be of lateral origin. (**C**) Laterally transferred regions are constructed from sets of nearby lateral *k*-mers, where nearby means separated by ≤gap *G*. For representation as a network, recipient sequences are subsumed into their respective groups with the result that transfers inferred from a donor group to a recipient sequence (**D**, left) are shown as from a donor group to a recipient group (**D**, right). For clique analysis, edge weight and directionality may further be ignored (see text).

The resulting workflow (Figure 4) differs from AF workflows for purely vertical phylogenetics (e.g. Figure 2) in two main ways: the unit of analysis is not specified up front, and sequences must be arranged into groups. Potential lateral segments are generated by merging *k*-mers that meet the IDF and TF requirements. A parameter *G* specifies the maximum allowable gap between *k*-mers to be merged into a lateral segment; where investigated, the number of LGT detections and total detection length were relatively insensitive to *G*. The resulting segments are typically of different lengths, and may map to intergenic regions, gene fragments, entire and/or multiple genes. In contrast, grouping the sequences in an effective manner proved to be non-trivial, yet critical to performance [33, 34, 99]. TF–IDF performs best when sequences are similar within group but dissimilar between groups; so if our goal is to infer LGT, the best grouping will probably capture hierarchical descent. Even so, it may remain ‘difficult to disentangle the effects of group number, size, composition and phylogenetic cohesion’ [34].

With simulated data, it was obvious how to delineate groups and TF–IDF performed well, as measured by precision and recall, over a biologically realistic range of sequence lengths and evolutionary distances between and within groups. As expected, greater evolution post-LGT had a deleterious effect on performance, while deletions showed relatively little effect. With empirical data, groups (e.g. γ-proteobacteria) known to engage in LGT were usually prominent in our TF–IDF analyses, while we inferred little or no LGT for groups known to be more quiescent [34]. Because only genes (not arbitrary regions) have gene ontology annotation, to study the functional implications of the inferred LGT, we mapped the inferred lateral segments to genes, using data-dependent length and overlap thresholds [34, 99]. For protein-coding genes, we might alternatively have asked whether the inferred lateral segments overlap regions that encode active sites or SCOP domains.

For groups related by a hierarchical tree, it may be possible to extract further information. If a genomic region is inferred to have received genetic material from two or more groups that are topologically adjacent on the tree, we might (depending on details) instead hypothesize that there had been a single transfer from a common ancestor of the donor groups. Imperfect overlap of the inferred lateral regions could be ascribed to the vagaries of subsequent evolution, and/or the IDF threshold being a blunt instrument. On the other hand, transfers from unrelated donor groups would render such a region an evolutionary mosaic [34]. Although gene loss and LGT among the donor lineages may present further complications, TF–IDF seems to promise a first-ever systematic look at the temporal dynamics of superposed transfers.

Summary information from TF–IDF analysis can be collected in the form of an LGT network. For simplicity of interpretation, recipient sequences are subsumed into their respective groups, and inferred transfer events consolidated as weights on the edges. Given the limitations of most clique-finding algorithms, the weights and directionality of edges are usually ignored [99]. Densely connected regions within an LGT graph—maximum cliques, maximal cliques and paracliques (cliques missing a few edges)—can be extracted using GrAPPA [100]. These structures demarcate genetic exchange communities (GECs), groups of taxa whose members have shared genetic material among themselves by LGT [101]. Taxa that retain membership across biologically reasonable values of *k* (for the examples cited, 20 ≤*k* ≤40) are considered core nodes of these GECs [99]. Other structures in an LGT graph may also be of biological interest, e.g. bridging nodes that connect cliques [102, 103]. By annotating nodes and edges with metadata, e.g. on environment, genome type or vector, new perspectives may be gained on the genetic structure of the microbial biosphere, and on genetic flow within and across ‘independent genetic worlds’ [102, 104–107].

The TF–IDF algorithm is scalable, running in $O\left(nL\cdot \log(nL)\right)$ time where *n* is the number of sequences and *L* their average length. Moreover, as the inferred edges are natively lateral and directional, computationally hard steps involving the generation of a reference topology and comparison with test trees are obviated. However, in its current implementation, TF–IDF is somewhat greedy of memory, preventing its application to very large data sets. Clique finding is computationally demanding, even though information on edge directionality and weight is typically ignored.

An analogous approach could be taken to identify regions of vertical inheritance. Wong and Ragan [108] recognized core regions that find matches in other sequences, extended these regions using a criterion of mutual exclusivity, built a pairwise similarity graph and applied MCL [109] to yield sets of putatively homologous subsequences they called Markov Clusters of Homologous Subsequences (MACHOS). In place of Smith–Waterman, match and extension criteria based on *k*-mers (above) could equally well be used. MACHOS correspond well to known Pfam domain families [108], and offer an AF approach to recognition of orthologs [110]. It has been argued that workflows in which a gene or protein family is, by default, considered to be inherited vertically unless this null hypothesis is specifically rejected gives a conceptually and methodologically unfair advantage to vertical inheritance [111, 112]. Doolittle [111] goes so far as to call this a ‘false null’. Parallel AF workflows for lateral and vertical regions could address this objection, inferring ‘LGT directly, positively and fairly in large genome-scale datasets’ [99].

## Conclusions

The power of *k*-mer-based AF approaches relies on proper selection of *k*. The requirement that *k*-mers be approximately unique to a sequence can be satisfied at a much smaller *k* for amino acids (alphabet size 20) than for nucleotides (alphabet size 4). For tree inference, optimal *k* depends on the length and divergence of the sequences, and (more weakly) on the inference method. In our hands, *k* is optimal at about 3–5 for proteins, and 8–10 for genes or RNAs [32, 63, 71]. We set *k* = 12 for a quick assessment of the relative divergence of microbial genome data sets [34, 73], while Greenfield and Roehm [31] used *k* > 15 to identify organisms, genes and functions of interest using unique *k*-mers as tags. For genome trees, optimal *k* ranged from 8 for isolates of the same bacterial species up to 25 across bacteria and archaea [63, 73]. Elhai *et al.* [92] used *k* = 8 to detect genes of recent lateral origin in microbial genomes, but needed to draw on additional lines of evidence to make their approach effective. In TF–IDF, optimal *k* is larger still, for microbial genome data sets in the range 25–40.

AF approaches are beginning to make their mark in phylogenomics and LGT research. Substrings can readily be extracted from sequences, indexed, stored and retrieved. They capture homology signal in evolving sequences, and counts or frequencies of shared *k*-mers can underpin measures of pairwise distance and the computation of distance trees. Distributions of *k*-mers among groups of genomes can reveal donor–recipient relationships in LGT, hence communities of genetic exchange, and may be informative on the temporal dynamics of reticulate evolution. Like their MSA-based counterparts, *k*-mer distance trees can be computed quickly and scale to very large data, without the computational overhead of a complex substitution model (or multiple such models for different sequence regions). Whether this is, on balance, a good thing remains to be seen, even apart from the question of whether it makes sense to infer a genome tree [113]. MSA-based methods have benefitted from more than four decades of development, in the process enriching all their component fields, biological and otherwise. In contrast, AF phylogenomics is still in its infancy. We anticipate that AF methods will mature to provide dependable options in large-scale phylogenomics while stimulating the exploration of other biological questions previously unimaginable within the classical framework.

##

**Key Points**

- Molecular sequences can be represented by sets of their constituent *k*-mers. To the extent that these *k*-mer sets capture the signal of homology among these sequences, they can inform on phylogenetic relationships.
- Measures of the intersections of these *k*-mer sets can be used, after normalization, to compute pairwise distances without the need for MSA.
- Trees computed from *k*-mer distances are often biologically reasonable, e.g. recovering recognized taxa, while being robust against evolutionary scenarios that are problematic for alignment-based phylogenetics.
- Using TF–IDF, it is possible to identify regions that have been transferred from a donor group into a recipient sequence. Across a data set, all such pairwise relationships describe an LGT network. Densely connected regions in LGT networks can be interpreted as GECs.
- These AF approaches are computationally fast and scalable to large nucleotide or amino acid data sets.
